# Transient Contact Elastic–Plastic Characteristics Analysis of Rail Welded Joints in Heavy-Haul Railways

**DOI:** 10.3390/ma19061246

**Published:** 2026-03-21

**Authors:** Chen Liu, Zhiqiang Wang

**Affiliations:** 1 School of Traffic and Transportation, Shijiazhuang Tiedao University, Shijiazhuang 050043, China; 2 State Key Laboratory of Rail Transit Vehicle System, Southwest Jiaotong University, Chengdu 610031, China; 3 State Key Laboratory of Mechanical Behavior and System Safety of Traffic Engineering Structures, Shijiazhuang Tiedao University, Shijiazhuang 050043, China; 4 Shanghai Key Laboratory of Rail Infrastructure Durability and System Safety, Tongji University, Shanghai 201804, China

**Keywords:** heavy-haul railway, welded joint, finite element method, contact stick-slip, elastic–plastic feature

## Abstract

**Highlights:**

**What are the main findings?**

- Contact state shifts from stick-slip to slip in welded zones, increasing wear susceptibility.
- Plastic deformation initiates at the rail surface due to stress–strain concentration at key damage sites.
- Parametric analysis assesses and ranks parameter effects on damage initiation.

**What are the implications of the main findings?**

- Guides welded joint material selection and parameter optimization for durability.
- Supports targeted rail surface maintenance and damage-prevention strategies.
- Informs operational adjustments (speed, mass, friction) to reduce plastic deformation.

**Abstract:**

This study investigates the transient wheel–rail contact mechanics of welded joints in heavy-haul rails via a validated 3D finite element model, and analyzes the stick-slip behavior, dynamic response and elastoplastic characteristics in the base material zone, heat-affected zone and weld bead zone. Results show a distinct contact state transition from stick-slip in the base material to predominant slip within the welded zones, indicating higher wear susceptibility. Dynamic response analysis reveals the highest and lowest contact-point acceleration amplitudes in the base material and heat-affected zone, respectively, due to material heterogeneity. Plastic deformation consistently initiates at the rail surface, where stress and strain concentrate, establishing it as the primary site for damage nucleation. A systematic parametric study shows that plastic deformation can be effectively mitigated by increasing the yield strength and elastic modulus of the welded joint material, or reducing the wheelset velocity, unsprung mass and wheel–rail friction coefficient. In contrast, adjusting the primary suspension and fastener parameters exerts a negligible influence on plastic deformation control. These findings provide a mechanistic basis for optimizing the performance and maintenance of welded joints in heavy-haul rail operations. This study reveals the coupling law of multiple mechanisms among contact behavior, dynamic response and material failure during the damage initiation process of rail welded joints from the mechanistic perspective, which provides a theoretical basis for the structural optimization, condition assessment and maintenance of rail welded joints in heavy-haul railways.

## 1. Introduction

With the continuous development of global trade integration and the rapid growth of logistics demand, heavy-haul railways, as the backbone of land transportation, exhibit increasingly prominent advantages of large transport capacity, high efficiency and low cost, and have thus become an inevitable trend of modern transportation systems. However, the improvement of transport capacity, particularly the significant increase in axle load and the rise in operating velocity, has brought unprecedented severe challenges to the service performance of track infrastructure. In the entire track structure, rail welded joints have always been regarded as the “weak links” in the track structure chain due to the discontinuities in metallographic structure, geometric form, and mechanical properties caused by their manufacturing processes (such as flash butt welding and thermite welding) [1]. When heavy-haul trains pass at high velocities, the wheel–rail interaction in the welded joint area manifests itself as an extremely complex dynamic process characterized by transience, high frequency, and high impact [2]. Severe dynamic impacts not only induce wheel–rail contact stress far exceeding that in conventional track sections, but also cause significant elastoplastic deformation, material fatigue damage, accelerated wear and typical defects such as joint sagging [3,4,5], which severely endangers train operation safety and significantly increases the maintenance costs of the track. Therefore, systematically investigating the contact mechanical behaviors and elastoplastic response characteristics of rail welded joints in heavy-haul railways under transient impact loads, and revealing their damage evolution mechanisms, is of crucial theoretical value and engineering practical significance for optimizing joint design, formulating scientific maintenance standards, extending the service life of rails, and ensuring the safety and economy of heavy-haul transportation.

Wheel–rail contact is the source of driving dynamic behaviors, and its transient characteristics in the welded joint area are particularly complex. Moreover, analyses related to contact characteristics mainly focus on two levels: the microscopic stick-slip behavior and the macroscopic dynamic response of the contact area. Stick-slip distribution characteristics directly determine the magnitude of creep force between wheel and rail as well as energy dissipation, and serve as the foundation for studying friction and wear, and rolling contact fatigue [6]. Under the influence of geometric irregularities in the joint area and variations in material hardness, the contact pressure distribution is no longer uniform, resulting in significant dynamic variations in the stick-slip distribution [7]. Particularly in curved track sections, the stick-slip distribution at the inner wheel–rail interface may exhibit periodic variations, which is regarded as one of the important causes of rail corrugation [7]. Key indicators of dynamic analysis include wheel–rail contact forces, rail displacements, and accelerations, which reach peak values instantaneously at the joints and have magnitudes far greater than the responses on continuous rails [8]. Field test data show that under heavy-haul conditions, when 25 t axle load freight cars pass through welded joints, the generated vertical rail forces increase by 17% to 18% compared with those of 21 t axle load locomotives, and the vertical accelerations increase by 12% to 17%. This fully quantifies the amplification effect of axle load on dynamic impacts [9]. The intense dynamic responses are the direct driving force leading to the fatigue damage accumulation of rails and substructure components, the aggravated plastic deformation in the welded joint area (sagging), and the failure of fastener systems.

The passage of heavy-haul trains through welded joints generates significant impact forces, such that the stress levels in the contact area easily exceed the yield limit of the rail material, thereby entering the plastic deformation stage. Therefore, conducting elastoplastic analysis is the key step for understanding damage mechanisms and predicting service life [10]. Due to the existence of the weld heat-affected zone, the material properties (such as hardness and yield strength) of different parts of the joint are not uniform, which makes the stress–strain distribution more complex, while plastic deformation tends to concentrate more easily in the softened zones with lower hardness [4]. Furthermore, to systematically evaluate the contributions of various influencing factors to the dynamic mechanical behaviors of welded joints and provide guidance for engineering applications, parametric studies are an indispensable component [11,12,13,14,15]. Parametric studies can identify the dominant factors affecting the service performance of welded joints and quantify their degrees of influence, thereby providing data support and theoretical guidance for the optimization of manufacturing processes for rail welded joints in heavy-haul railways, the scientific formulation of track maintenance standards, and the development of new high-strength and toughness welding technologies.

Performance evolution of rail welded joints is directly pivotal to the normal operation of heavy-haul railways, and clarifying the mechanical behavior characteristics of their joint zones under service conditions constitutes an essential prerequisite for ensuring the long-term reliable operation of heavy-haul railway lines. With the development of modern railway systems toward higher efficiency, greater light-weighting and enhanced intelligence, lightweight composite materials have been increasingly applied in vehicle structures, which presents multi-dimensional challenges for the service performance assessment of railway infrastructure under novel dynamic load spectra [16,17]. In response to the aforementioned issues, this study aims to go beyond the superficial description of the damage phenomena of rail welded joints and probe deeply into and reveal the underlying failure mechanisms involving multi-physical field coupling. This study focuses on the spatiotemporal coupling laws among the dynamic evolution of the local stress–strain field, the instability of the interfacial contact state, and the initiation of early-stage plastic damage in rail welded joints caused by material and geometric inhomogeneities under heavy-haul conditions, and clarifies how this coupling mechanism quantitatively governs the origination and initial evolution of damage. Accordingly, this study focuses on investigating the transient contact mechanical behavior of the rail welded joint zone in heavy-haul railways via the finite element method. First, on-site investigations are carried out on typical heavy-haul railway lines to clarify the distribution and damage forms of rail welded joints, and a 3D coupled finite element model of the vehicle wheelset and track is established based on the actual operating conditions of the investigated section, with the welded joint area modeled in detail. Second, the wheel–rail contact characteristics (stick-slip distribution and dynamic response) are analyzed using the validated numerical model. Third, the elastoplastic characteristics of the welded joint under wheel–rail contact are explored, including the time-varying and spatial distribution features of stress and strain. Finally, a systematic parametric analysis is performed to quantify the action mechanism of various influencing parameters on the mechanical properties of the wheel–rail-track coupled system.

## 2. On-Site Investigation and Model Establishment

This section elaborates on the on-site and operating conditions of the straight section of heavy-haul railways with rail welded joints, and focuses on the morphological characteristics and damage forms of the welded joints. Based on the actual operating conditions of the investigated section, finite element technology was adopted to establish a coupled numerical model of wheelset and track suitable for heavy-haul railway scenarios, so as to lay a foundation for the subsequent analysis.

### 2.1. On-Site Investigation

**Figure 1:**
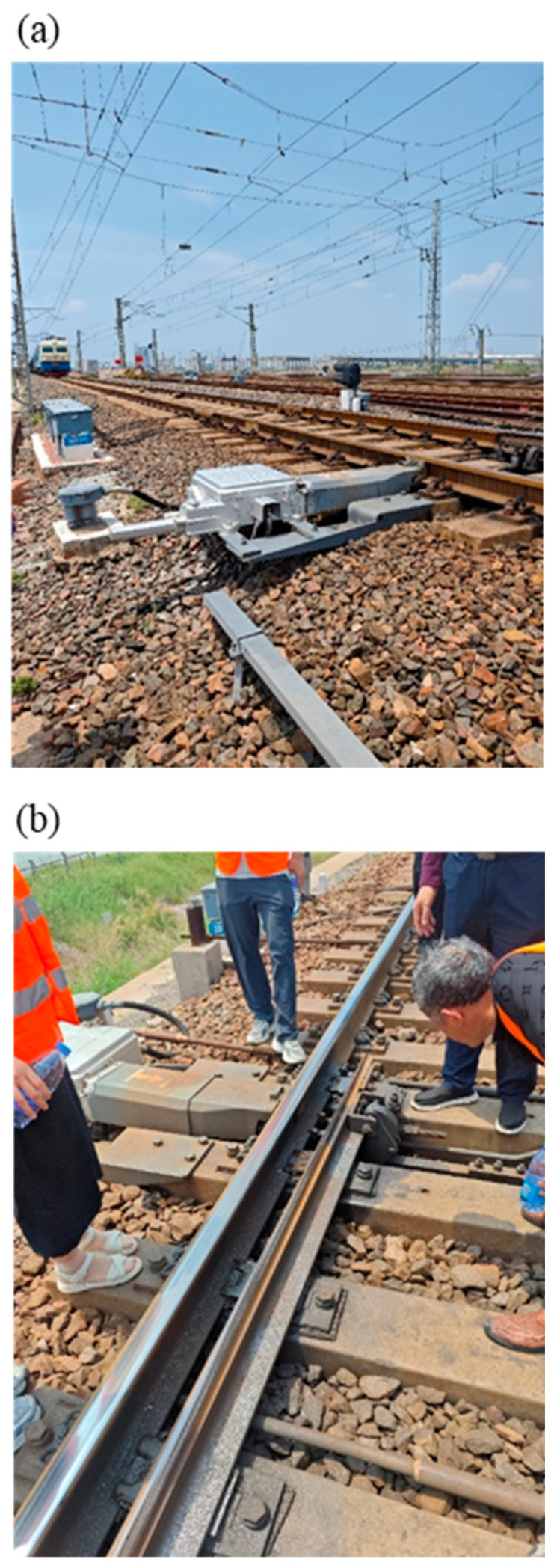
Field pictures of the research section. (**a**) Field picture 1, (**b**) Field picture 2.

The actual investigated section is located in the straight section of Handan-Huanghua Railway, which is classified as Class I national railway with a designed velocity of 120 km/h, and the vehicle operating velocity in the investigated section is 80 km/h. The entire line adopts 100 m fixed-length new rails with a weight of 60 kg/m, and forms continuous welded rail through welding and laying. The main type of sleepers adopted is Type III shouldered concrete sleepers, with a laying density of 1667 pieces per kilometer; while in special bridge sections, new Type III bridge sleepers are appropriately adopted as needed. The fastener systems generally adopt Type II elastic clip fasteners, while low-resistance fasteners are used in some special sections to adapt to changes in track longitudinal forces. The ballast bed structure adopts ballasted track, and the ballast bed material is Class I crushed stone ballast. The locomotive type is the HXD3 electric locomotives, with an axle load of 23 t and a designed traction mass of up to 5000 t. On-site photographs of the investigated section are shown in Figure 1.

**Figure 2:**
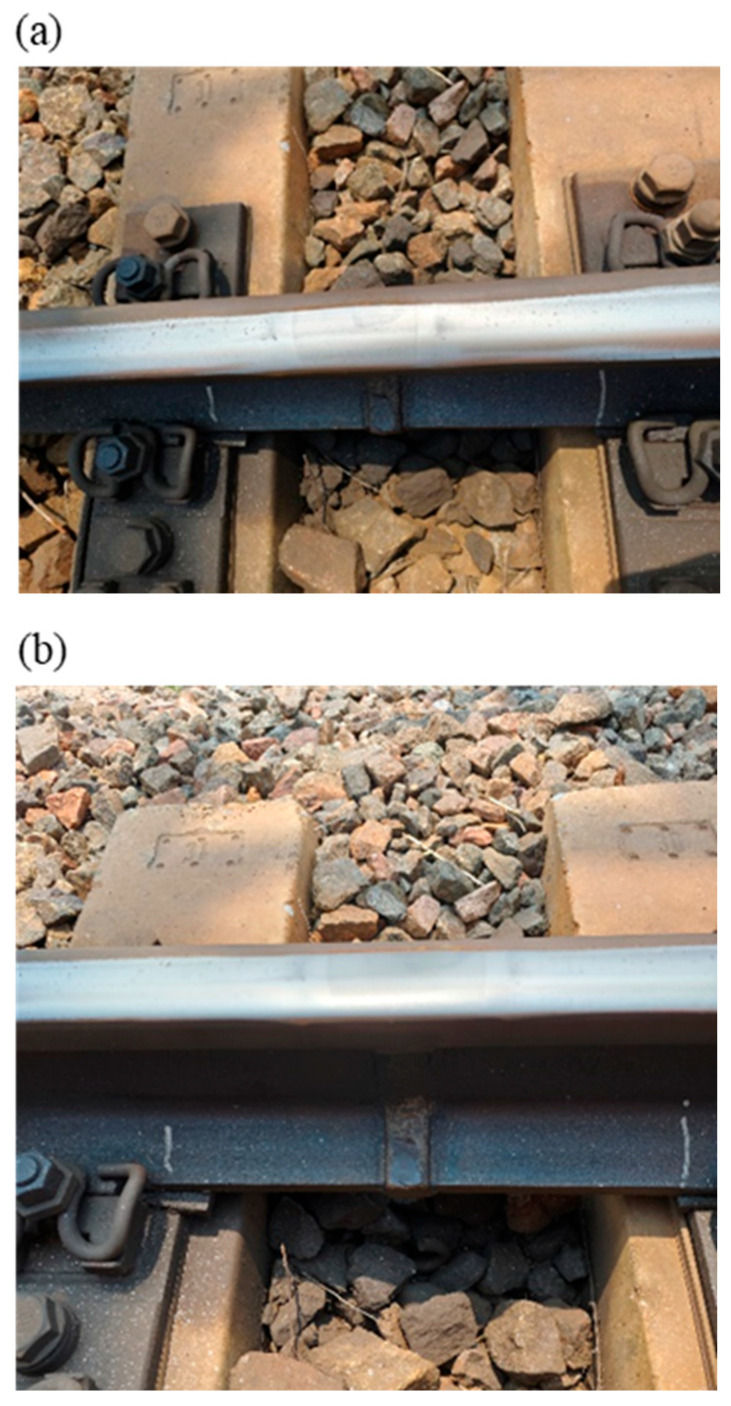
Pictures of rail welded joints. (**a**) Welded joint 1, (**b**) welded joint 2.

**Figure 3:**
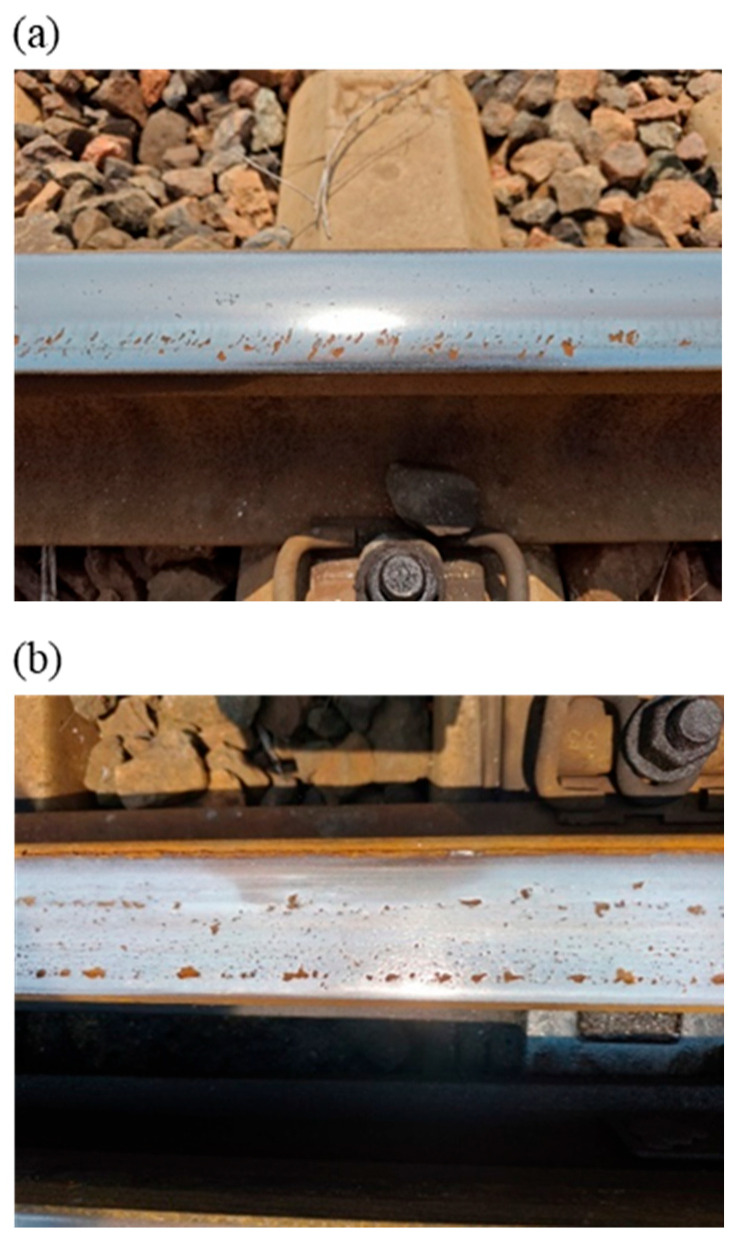
Field pictures of rolling contact fatigue and peeling. (**a**) Rail surface damage picture 1, (**b**) rail surface damage picture 2.

Within the actual investigated section, there are multiple flash butt welded joints on the track, as shown in Figure 2. As can be seen from Figure 2, there are obvious welded bead zones, heat-affected zones, and base material zones in the joint area, and the welded bead zone and heat-affected zone vary in length. Welded joints impair the flatness of the rail surface to a certain extent and introduce excitation sources into the dynamic response of the wheel–rail system. Thus, near the welded joint area, structural damages are prone to developing on the rail surface, such as rolling contact fatigue and material spalling and chipping, as shown in Figure 3.

As can be seen from Figure 3, severe rolling contact fatigue cracks and material spalling and chipping have occurred on the rail surface. The starting ends of the fatigue cracks are approximately parallel to the rail cross-sectional direction, while their terminal ends are approximately at a 30° angle to the rail cross-sectional direction, and induce material spalling and chipping, as shown in Figure 3a. Under the action of wheel impact and cyclic loads, some rail surfaces exhibit large-scope material spalling and chipping, accompanied by widespread pitting damage, as shown in Figure 3b. Additionally, non-uniform wear phenomena such as rail surface pits and rail corrugation also exist near the joint area, but they are relatively mild in degree. The formation mechanisms of the aforementioned damages will be analyzed in detail in subsequent sections. The observed surface damage, especially the cracks and spalling shown in Figure 3, is closely associated with the inhomogeneous mechanical responses in the rail welded joint zone. This study focuses on investigating the contact mechanical behavior and elastoplastic response of different subzones (the base material zone, the heat-affected zone and the weld bead zone) of rail welded joints under transient wheel–rail impact, aiming to reveal the mechanical conditions and spatial locations where such surface defects preferentially initiate from a mechanistic perspective.

### 2.2. Model Establishment

**Figure 4:**
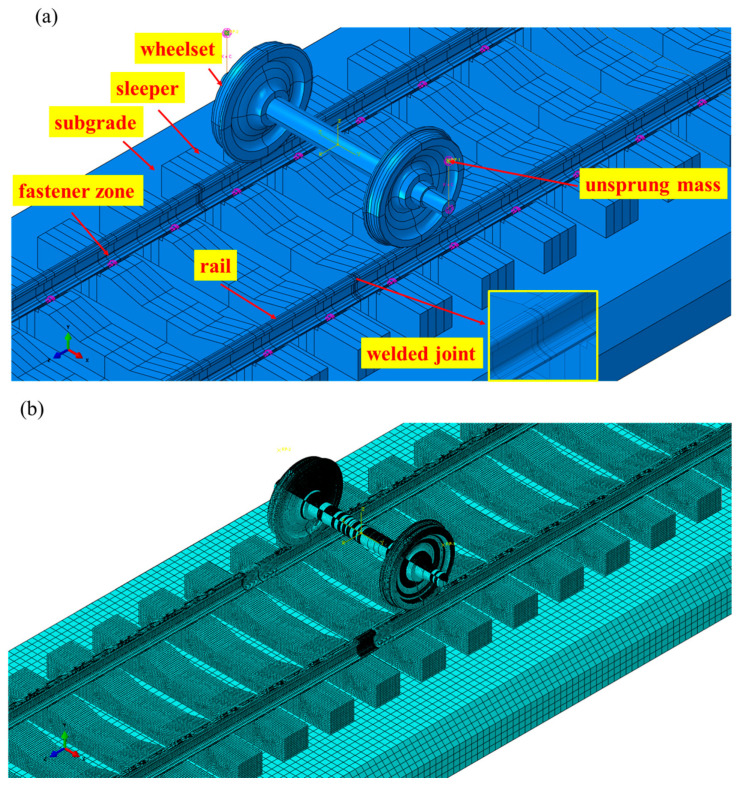
Numerical model. (**a**) Model structure diagram, (**b**) model mesh diagram.

Based on the actual investigated railway section, a 3D coupled finite element model of the wheelset and track is established to simulate the heavy-haul railway wheel–rail interaction, as shown in Figure 4. In this model, the unsprung mass is modeled as a single mass point of 11.5 t, which is connected to the wheel axle center via spring-damping elements (i.e., the primary suspension) to simulate the wheels of heavy-haul railway vehicles. The numerical model has a longitudinal length of 12 m and includes components such as the wheelset, rails, sleepers, and subgrade, where the wheel profile is LM_B_ and the rail profile is CN60. The rails and sleepers are connected via fasteners, which are simulated using spring-damping elements, and the sleepers are fixed to the subgrade. A rail welded joint is introduced in the middle region of the rail, with a weld width of 18 mm and a width of 18 mm for the heat-affected zone on each side. The different subzones are primarily distinguished by material parameters, which aim to accurately characterize the inherent material property gradient of the joint. Characterization of such inhomogeneity serves as the physical basis for capturing abrupt changes in the local mechanical field and further investigating the spatial selectivity of damage initiation, rather than being a mere geometric refinement. The material and structural parameters of the model are listed in Table 1 [18,19,20,21,22,23]. The material parameters for each subzone of the welded joint in the model of this study are integrated with the typical mechanical property test data of flash butt welded joints of U75V rails reported in the existing literature, and thus represent the typical values for such joints. The model is intended to reveal the response differences and their underlying mechanisms in the welded joint zone under transient impact. Its qualitative predictive capability can be cross-validated by correlating the damage mechanisms revealed by simulations with the widely observed field damage morphologies (see Section 3 and Figure 3).

In the numerical model, the wheel–rail interface is coupled through contact, where the normal behavior is described using “hard contact” and allows separation of the wheel and rail after contact. The tangential behavior is described using the penalty function friction formula, with the friction coefficient set to 0.45 [24]. In the 3D wheel–rail rolling contact finite element model, the distinction between adhesion zones and slip zones in the contact area can be achieved by setting a friction force threshold [25,26,27,28]. In the adhesion zones, the nodal friction force $F_{i}$ can be expressed as:

(1) $$F_{i}=k\Delta \gamma_{i}$$

In the formula, k is the adhesion stiffness; $\gamma_{i}$ is the nodal relative slip displacement, and $\Delta \gamma_{i}$ is the variation in the nodal relative slip displacement. When i takes the values of 1 and 2, they represent the longitudinal and lateral directions respectively. In the slip zones, the nodal friction force can be expressed as:

(2) $$F_{i}=F_{t}\frac{\Delta \gamma_{i}}{\Delta \gamma }$$

(3) $$F_{t}=\mu p$$

(4) $$F_{f}=\sqrt{F_{1}^{2}+F_{2}^{2}}$$

In the formula, $F_{t}$ is the friction force threshold; γ is the total nodal relative slip displacement, and Δγ is the variation in the total nodal relative slip displacement; μ is the friction coefficient; p is the nodal normal contact stress; $F_{f}$ is the resultant nodal friction force. When $F_{f}=0$, the contact point is in a non-contact state; when $0<F_{f}<F_{t}$, the contact point is in an adhesion state; when $F_{f}\geq F_{t}$, the state of the contact point transitions from adhesion to slip.

**Figure 5:**
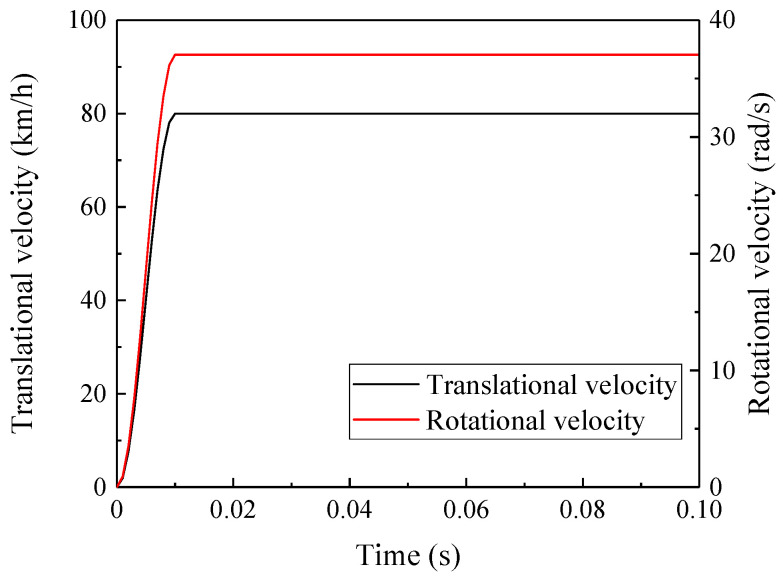
Velocity curves.

Wheel–rail rolling contact simulation is achieved by setting the translational velocity and rotational velocity of the wheels. Due to the fact that the instantaneous application of velocities will induce significant dynamic interactions in the wheel–rail system, sinusoidal loading function curves are used to apply the velocities to mitigate the above-mentioned effect, as shown in Figure 5. The sinusoidal loading function can be expressed as:

(5) $$V_{T}\left(t\right)=\left\{\begin{array}{l} \begin{array}{cc} \frac{V_{0}}{2}\left(1-\cos\left(\frac{\pi t}{t_{0}}\right)\right), & t<t_{0} \end{array}\\ \begin{array}{cc} V_{0,} & t\geq t_{0} \end{array} \end{array}\right.$$

(6) $$\Phi_{T}\left(t\right)=\left\{\begin{array}{l} \begin{array}{cc} \frac{\Phi_{0}}{2}\left(1-\cos\left(\frac{\pi t}{t_{0}}\right)\right), & t<t_{0} \end{array}\\ \begin{array}{cc} \Phi_{0,} & t\geq t_{0} \end{array} \end{array}\right.$$

In the formula, $V_{T}\left(t\right)$ and $\Phi_{T}\left(t\right)$ are respectively the functions of translational velocity and rotational velocity with respect to time, with units of km/h and rad/s, and satisfy the relation $V_{T}\left(t\right)=0.0036\Phi_{T}\left(t\right)R$. R is the nominal rolling circle radius of the wheel, with a value of 600 mm; $V_{0}$ and $\Phi_{0}$ are the maximum translational velocity and maximum rotational velocity, with values of 80 km/h and 37.037 rad/s respectively; and $t_{0}$ is the initial time at which the translational velocity and rotational velocity reach their maximum values, with a value of 0.01 s. Additionally, a vertical coupled constraint is applied between the mass point simulating the unsprung mass and the wheel axle center to ensure that the two have consistent motion states.

The boundary conditions of the numerical model are specifically as follows: the longitudinal end faces of the track structure are provided with longitudinal symmetric constraints, the bottom surface of the subgrade is provided with fixed constraints, and no constraints are applied to other parts. In the simulation process, a total of two implicit calculation analysis steps are set. The first step is the wheel–rail contact static calculation, which is subjected to gravity loads and unsprung mass loads. The second step is the wheel–rail rolling contact dynamic calculation, which applies the velocity function curve. To simulate the longitudinal slope characteristics of heavy-haul railways, a local coordinate system is set during the model establishment process to incorporate the track longitudinal slope gradient. To balance the computational efficiency and the analysis accuracy of key regions, the model adopts a non-uniform meshing strategy. The mesh in the welded joint area of the model is refined. Specifically, the mesh size of the rail surface in the welded bead zone and the heat-affected zone is 2 mm × 2 mm × 2 mm; the mesh size of the rail surface in the base material zones on both sides of the heat-affected zone is 2 mm × 4 mm × 2 mm; and the mesh size of other rail parts is controlled to gradually transition to an approximate global mesh size of 20 mm. The mesh size of the wheel tread contact area is 2 mm × 2 mm × 2 mm, while the mesh size of other wheel parts is controlled to gradually transition to an approximate global mesh size of 20 mm; the global mesh size of the sleeper mesh is 20 mm. The global mesh size of the sleeper mesh is 20 mm; the global mesh size of the subgrade mesh is 80 mm. The selected mesh density matches the scale of the heavy-haul wheel–rail contact patch, enabling effective characterization of the contact pressure gradient. It is also consistent with the commonly adopted resolution in similar elastoplastic contact finite element studies, and can thus meet the research objective of analyzing the distribution trend of macroscopic plastic strain [6]. The entire numerical model is meshed with two types of elements, C3D6 and C3D8R, with a total of 1,641,567 nodes and 1,465,542 elements. The mesh diagram of the model is shown in Figure 4b.

The model in this study assumes that the initial stress state of the rail and its welded joint prior to the application of wheel–rail loads is zero. This assumption helps to decouple the independent effect of external impact loads, thereby more clearly revealing the fundamental influence of material and geometric inhomogeneities on the transient mechanical response. For the material constitutive behavior, the bilinear isotropic hardening model is adopted to describe the elastoplastic behavior of rail welded joints. This model can effectively characterize the initial yielding and subsequent hardening response of the material under monotonic or finite-number high-amplitude impacts, and is suitable for revealing the relative trend and spatial distribution of plastic deformation initiation in each subzone of the welded joint during single or initial-stage wheel–rail impacts. This choice strikes a reasonable balance between computational efficiency and the core objective of this study focusing on the initial mechanism of damage initiation.

## 3. Contact Characteristics Analysis

This section primarily focuses on conducting research on the contact characteristics of the rail welded joint area in heavy-haul railways. By utilizing the finite element model established in Section 2.2, it analyzes the wheel–rail contact stick-slip distribution characteristics and dynamic response characteristics respectively, with the aim of clarifying the transient contact dynamic characteristics of the corresponding wheel–rail system and the formation mechanisms of relevant structural damages.

### 3.1. Stick-Slip Distribution Characteristics

**Figure 6:**
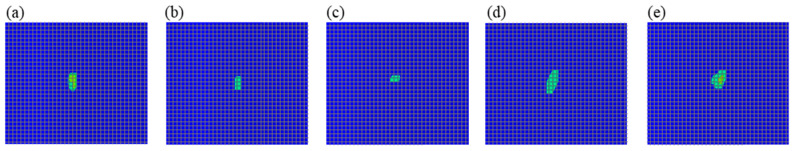
Distribution nephograms of contact stick-slip (the blue indicates no contact, green indicates slip, and yellow indicates adhesion). (**a**) Moment 1, (**b**) Moment 2, (**c**) Moment 3, (**d**) Moment 4, (**e**) Moment 5.

By utilizing the wheelset-track coupled finite element numerical model established in Section 2.2, the wheel running velocity is set to 80 km/h, the track longitudinal slope gradient is 20‰, the analysis step size is 0.0001 s, and the dynamic simulation calculation is performed. By extracting the stick-slip distribution nephograms of the contact patches in the base material zone, heat-affected zone, and welded bead zone of the rail welded joint, the relative motion states of the wheel and rail can be effectively determined. The corresponding nephograms are shown in Figure 6.

In Figure 6, Time 1 and Time 5 correspond to the stick-slip distribution nephograms of the contact patches in the front base material zone and rear base material zone respectively; Time 2 and Time 4 correspond to the stick-slip distribution nephograms of the contact patches in the front heat-affected zone and rear heat-affected zone respectively; and Time 3 corresponds to the stick-slip distribution nephograms of the contact patches in the welded bead zone. From Figure 6, it can be observed that when the wheel passes through the rail welded joint area, the wheel–rail contact first transitions from the stick-slip state in the base material zone to the slip state in the heat-affected zone and welded bead zone, and then transitions back to the stick-slip state. Since the wheel is in rolling motion when in the stick-slip state, while it is in slip motion when in the slip state, the wheel thus undergoes stick-slip motion in the rail welded joint area. Furthermore, the occurrence of slip zones indicates that the surface materials tend to undergo wear [29,30,31,32], and the wear range in the heat-affected zone and welded bead zone is relatively larger than that in the base material zone (as shown in Figure 6, the slip range in the heat-affected zone and welded bead zone is relatively larger than that in the base material zone). From the perspective of contact mechanics, this qualitatively explains why more severe wear, spalling, and rolling contact fatigue damage are frequently observed in the welded joint zone (especially the heat-affected zone and weld bead zone) during field observations (see Figure 3). The mechanism by which this inhomogeneous contact state leads to the tendency of inhomogeneous wear is consistent with common phenomena observed in engineering practice. Meanwhile, a comprehensive analysis of the stick-slip state transition in Figure 6 reveals that the heat-affected zone exhibits the most significant contact state instability. The proportion of the slip region within its contact patch is relatively larger, indicating that this region is more prone to micro-slip during wheel–rail interaction. This slip-dominated contact characteristic is one of the key factors that accelerate wear and initiate fatigue damage on the material surface.

### 3.2. Dynamic Response Characteristics

**Figure 7:**
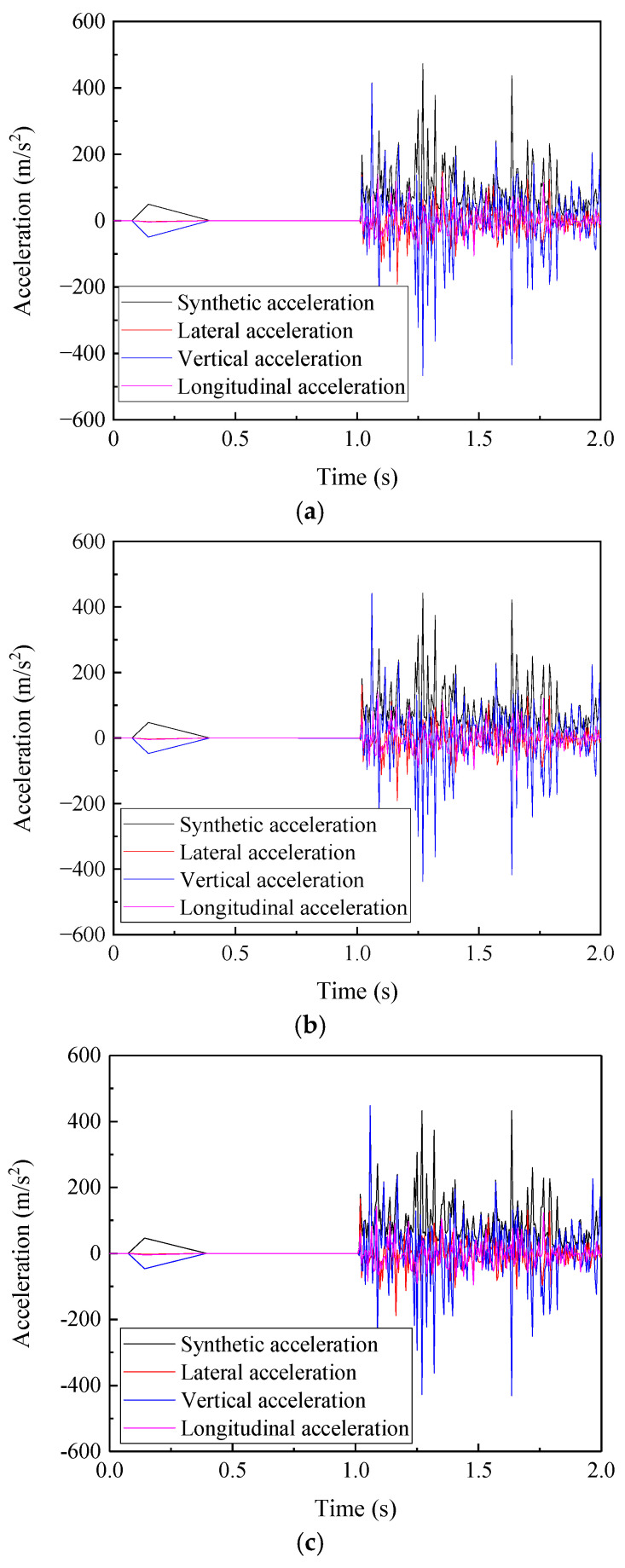
Acceleration curves at contact nodes in the welded joint region. (**a**) Acceleration curves at the contact node in the base material zone, (**b**) acceleration curves at the contact node in the heat-affected zone, and (**c**) acceleration curves at the contact node in the welded bead zone.

With the simulation conditions in Section 3.1 kept unchanged, dynamic calculations are performed using the finite element model. The acceleration data of contact nodes in the base material zone, heat-affected zone, and welded bead zone are extracted respectively, which are plotted in Figure 7.

From Figure 7, it can be observed that the acceleration curves of the three different zones of the rail welded joint all exhibit a certain degree of fluctuation characteristics, and the forms of the three acceleration curves are similar. The maximum acceleration of the contact nodes in the base material zone is 473.08 m/s^2^; the maximum acceleration of the contact nodes in the heat-affected zone is 442.57 m/s^2^; and the maximum acceleration of the contact nodes in the welded bead zone is 448.19 m/s^2^. From the comparison, it can be concluded that the acceleration amplitude of the contact nodes in the base material zone is the largest, the acceleration amplitude of the contact nodes in the welded bead zone is the second highest, while the acceleration amplitude of the contact nodes in the heat-affected zone is the smallest, and this is closely related to the material properties of the zones where the three are located. Since the acceleration amplitude of the contact nodes in all three zones of the rail welded joint exceeds 440 m/s^2^, the vibration intensity at the contact interface is relatively high during wheel operation. Based on the results of the stick-slip distribution calculation in Section 3.1, where slip contact occurs in both the heat-affected zone and the welded bead zone, it can be concluded that unstable vibration occurs at the contact interface, meaning the wheel–rail system exhibits an instability phenomenon [33,34,35,36]. System instability will induce the generation of initial damage on the wheel–rail surfaces. With the reciprocal operation of vehicle wheels, the initial damage will gradually evolve into obvious structural damage, such as rolling contact fatigue and peeling shown in Figure 3. Although the acceleration amplitude at the contact points of the heat-affected zone is relatively the lowest, this is associated with the reduced elastic modulus of the material and the enhanced cushioning effect. Combined with its significant slip trend in Section 3.1, this indicates that this region is in a unique mechanical environment where material softening is coupled with contact instability. This coupled state is highly likely to serve as a hotbed for damage initiation and requires further clarification combined with stress–strain analysis.

## 4. Elastic–Plastic Characteristics Analysis

Based on the finite element numerical model, this section conducts a study on the elastic–plastic characteristics of the rail welded joint area; on the one hand, it analyzes the time-varying characteristics of stress and strain at contact nodes in different regions of the welded joint. On the other hand, it analyzes the stress–strain distribution features of the cross-sections where the contact nodes are located (referred to as spatial features in subsequent sections), so as to understand the mechanical behavior of materials in the rail welded joint area.

### 4.1. Stress–Strain Time-Varying Features

**Figure 8:**
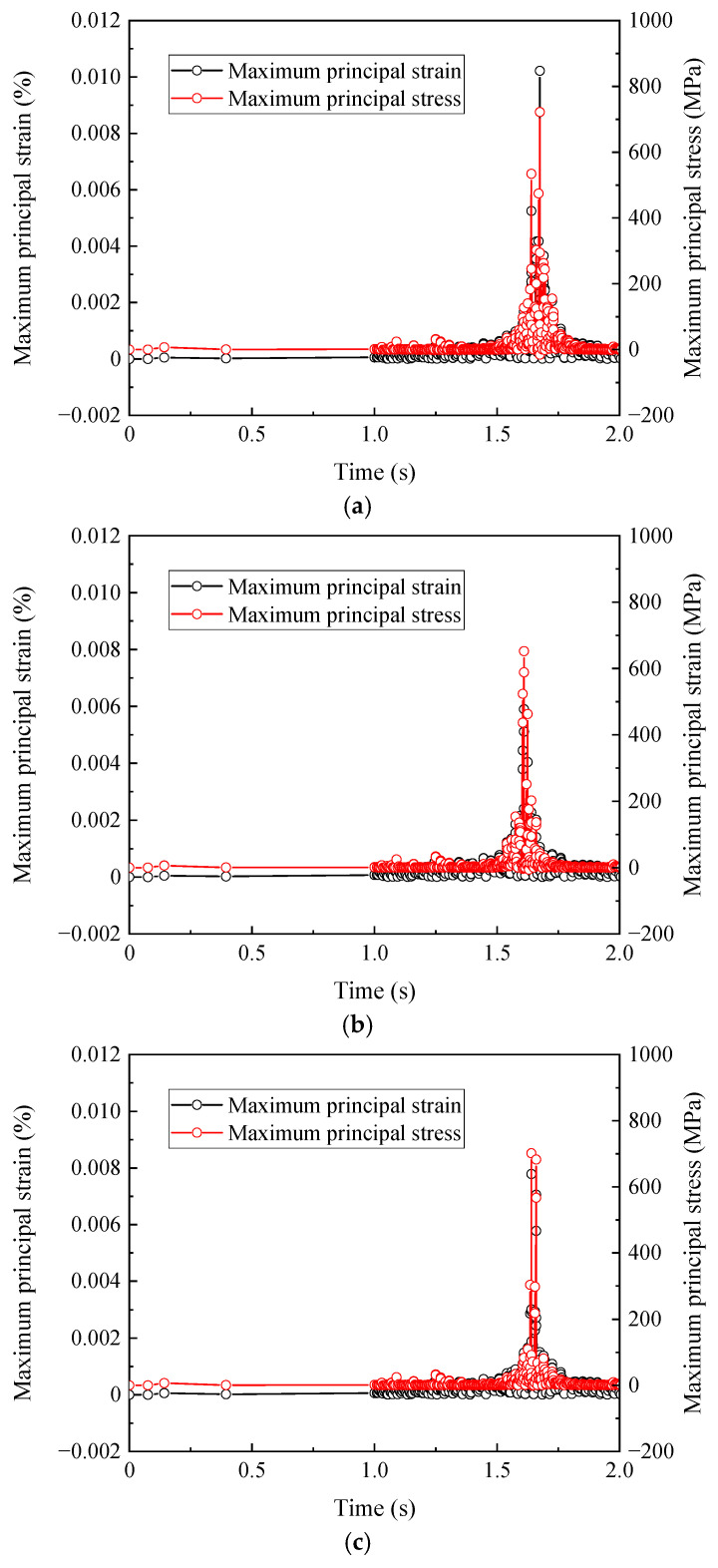
Stress–strain curves at contact nodes in the welded joint region. (**a**) Stress–strain curves at the contact node in the base material zone, (**b**) stress–strain curves at the contact node in the heat-affected zone, and (**c**) stress–strain curves at the contact node in the welded bead zone.

With the simulation conditions consistent with those in Section 3.1 set, dynamic simulations are performed using the numerical model. The maximum principal stress and maximum principal strain data of contact nodes in the base material zone, heat-affected zone, and welded bead zone of the rail welded joint are extracted respectively and plotted, as shown in Figure 8.

**Figure 9:**
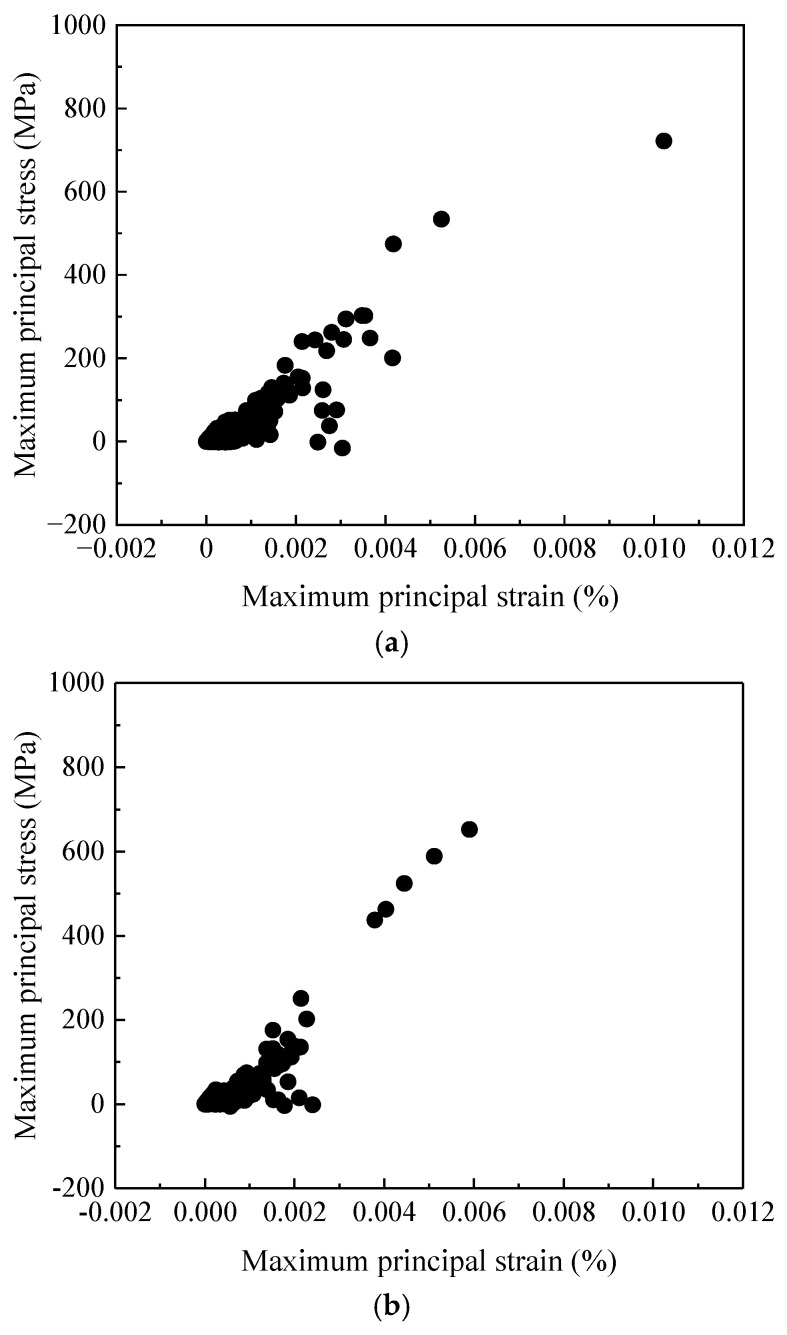

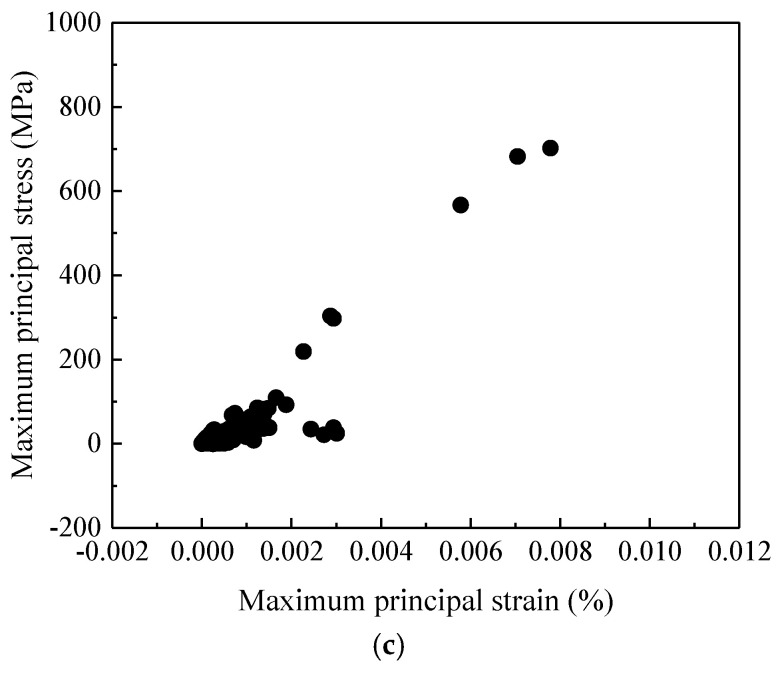
Stress–strain scatter diagrams at contact nodes in the welded joint region. (**a**) Stress–strain scatter diagram at the contact node in the base material zone, (**b**) stress–strain scatter diagram at the contact node in the heat-affected zone, and (**c**) stress–strain scatter diagram at the contact node in the welded bead zone.

From Figure 8, it can be observed that in the rail welded joint area, the maximum principal strain of the contact nodes in the base material zone reaches 0.012%, and the maximum principal stress reaches 746.90 MPa, which is close to the yield strength of 752 MPa for the base material. The maximum principal strain of the contact nodes in the heat-affected zone reaches 0.006%, and the maximum principal stress reaches 653.81 MPa, which is close to the yield strength of 655 MPa for the heat-affected zone material. The maximum principal strain of the contact nodes in the weld bead zone reaches 0.008%, and the maximum principal stress reaches 701.67 MPa, which is close to the yield strength of 702 MPa for the weld bead zone material. As described in Section 2.2, the yield strength and elastic modulus of the heat-affected zone are significantly lower than those of the base material. The preset material softening causes this zone to yield earlier and present a higher plastic strain concentration under the same impact load, which mechanistically explains why it acts as the preferential site for damage initiation. It can be seen from this that, under the aforementioned operating conditions, the material in the heat-affected zone has repeatedly entered or approached the critical yield state. This mechanical condition where cyclic stress reaches the bearing limit of the material serves as the direct prerequisite for plastic strain accumulation and fatigue crack initiation. In contrast, although the stress in the base material zone is higher, there is still a small margin from its yield limit, while the weld bead zone lies between the two. Therefore, the heat-affected zone is the first among the three zones to meet the critical stress conditions for defect initiation. This can also be observed from the stress–strain scatter diagram at contact nodes in the three zones, as shown in Figure 9.

### 4.2. Stress–Strain Spatial Features

**Figure 10:**
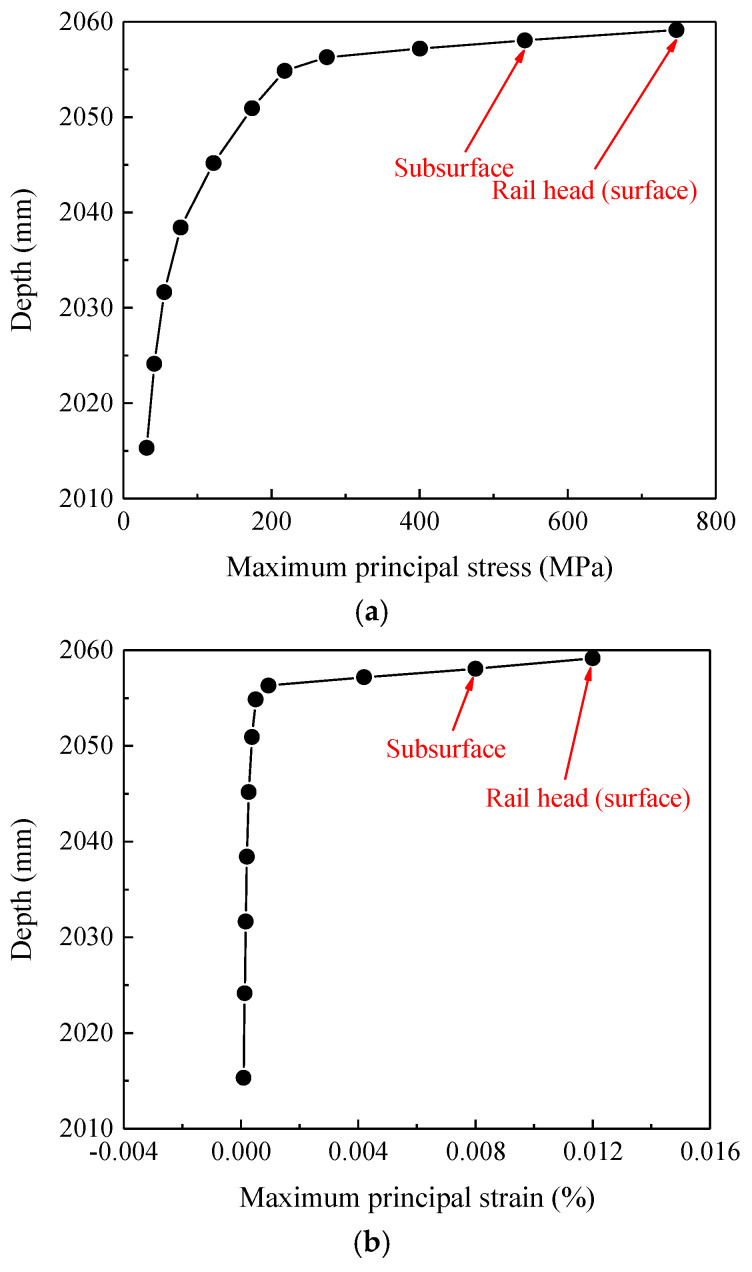
Stress and strain variation curves in the base material zone. (**a**) Stress variation curve, (**b**) strain variation curve.

**Figure 11:**
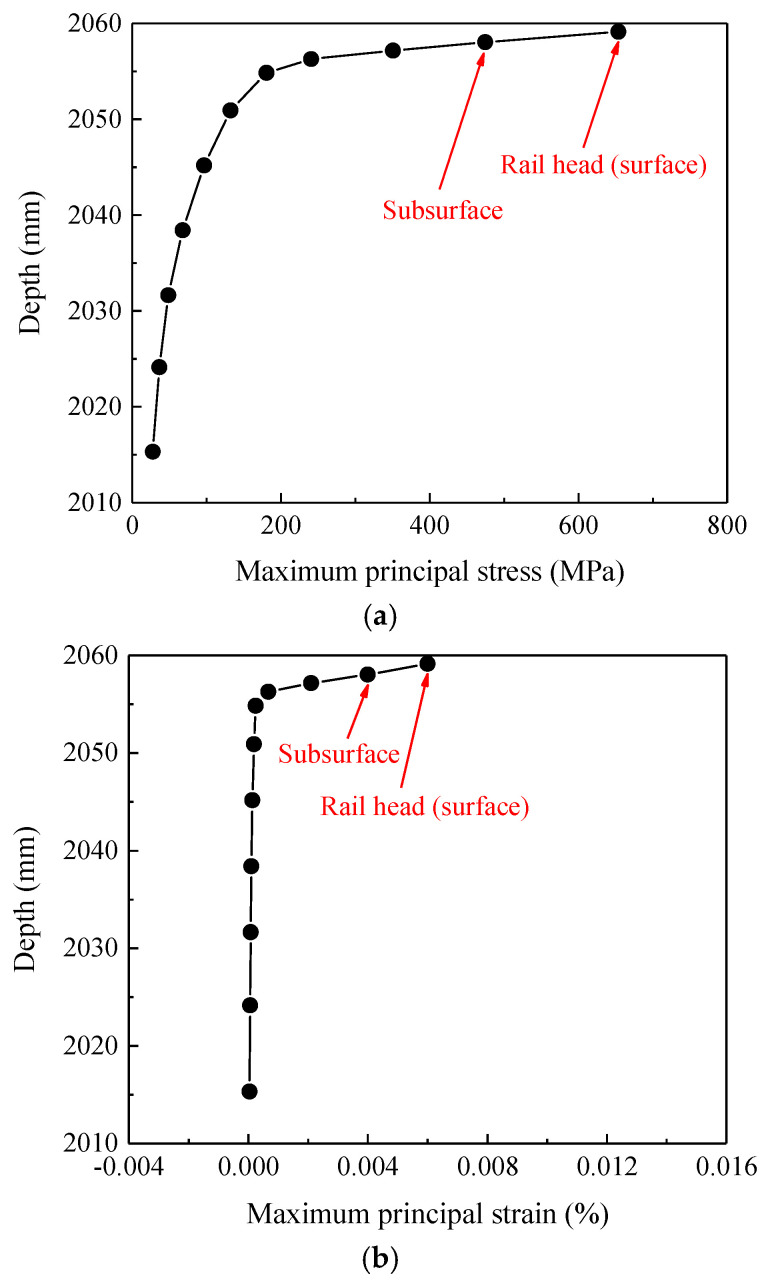
Stress and strain variation curves in the heat-affected zone. (**a**) Stress variation curve, (**b**) strain variation curve.

**Figure 12:**
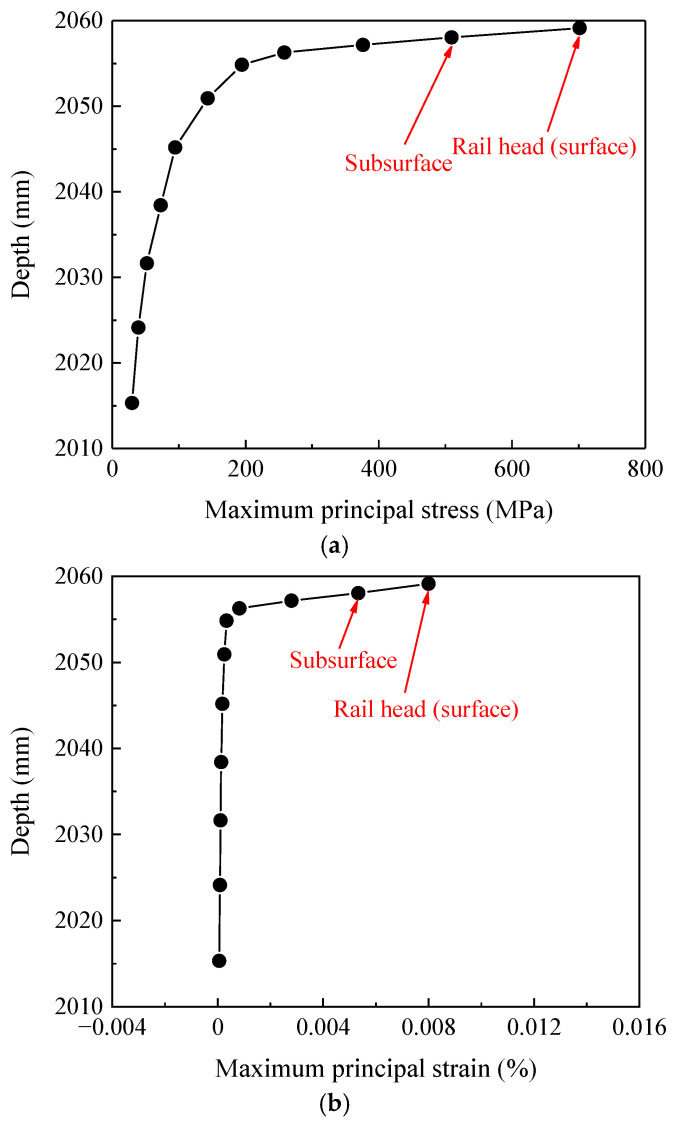
Stress and strain variation curves in the welded bead zone. (**a**) Stress variation curve, (**b**) strain variation curve.

On the basis of the analysis of the stress–strain time-varying features of contact nodes in the rail welded joint area, this section further conducts an analysis of the stress–strain spatial features of the sections where the contact nodes are located. Through numerical calculations, the variation curves of stress–strain with section depths in the base material zone, heat-affected zone, and welded bead zone can be obtained, as shown in Figure 10, Figure 11 and Figure 12.

The curves of stress–strain varying with the depth of the sections where the contact nodes are located, as shown in Figure 10, Figure 11 and Figure 12, indicate that for all three zones (the base material zone, heat-affected zone, and welded bead zone), both the maximum principal stress and maximum principal strain of the sections where the contact nodes are located are observed on the surface; additionally, the maximum principal stress and maximum principal strain at the subsurface are also relatively large. Meanwhile, as shown in Figure 10, Figure 11 and Figure 12, as the depth of the sections gradually decreases (the distance from the rail head gradually decreases), the maximum principal stress and maximum principal strain in the three zones of the rail welded joint first decrease sharply, then the rate of decrease slows down, and finally the stress and strain tend to stabilize. This distribution law, in which stress–strain decays rapidly from the surface layer to the interior spatially and tends to stabilize, is consistent with the basic knowledge of elastic–plastic mechanics. Moreover, the contour plot is continuous and smooth without abnormal oscillations caused by mesh discretization, indicating that the current mesh can reasonably capture the macroscopic gradient and also reflecting that the material stress–strain variations near the rail top are relatively large under wheel load.

Since material failure is closely related to the locations of the maximum principal stress and maximum principal strain, structural damage of the welded joint is more prone to occur in the rail surface/subsurface region. For the rail welded joint zone investigated in this paper, both the maximum principal stress and maximum principal strain appear on the rail surface. Therefore, the probability of damage initiation from the contact surface is significantly higher than that at other locations, which is consistent with field investigation results and indicates that the model can accurately capture the key location characteristics of damage initiation. Although the model results do not directly present macroscopic crack morphology, they have clearly revealed the specific location where defects are most likely to initiate, i.e., the surface layer of the heat-affected zone, and clarified the multi-field coupled mechanical inducements. Quantitative analysis further indicates that the surface and subsurface layers of the rail head in the welded joint are the most critical regions for stress–strain concentration and plastic deformation initiation under wheel–rail impact loads. This finding mechanistically explains the phenomenon observed in Figure 3 that damages such as rolling contact fatigue cracks and material spalling all initiate from the rail surface, thereby preliminarily identifying the potential initiation zone in the fatigue damage evolution process.

It should be noted that the above analysis on the initiation tendency and location of plastic deformation is derived from the material response under single or limited impact load cycles, which reveals the possible initiation sites and mechanical conditions of damage. For the prediction of accumulated plastic deformation (ratchet strain) and the final shakedown state of joints under long-term cyclic loading, more advanced cyclic plasticity constitutive models should be adopted for further investigation.

## 5. System Parametric Analysis

To evaluate the influence degrees of various system parameters on the initial mechanical properties of materials in the rail welded joint zone, a parametric study of the coupled system is carried out in this section. The objective of the parametric study in this research is to identify the key factors affecting the damage initiation tendency, so as to provide a basis for defect prevention through optimal design and operation. Using the control variable method, a total of 17 parameters are selected as analysis variables, including the yield strength and elastic modulus of materials in the base material zone (BMZ), heat-affected zone (HAZ), and the welded bead zone (WBZ), as well as wheelset velocity, unsprung mass, interface friction coefficient, primary suspension connection stiffness/damping, and fastener triaxial stiffness/damping. The maximum principal stress and maximum principal strain in the rail welded joint area are selected as the analysis objectives. By conducting multiple parameter-varying calculations, the specific mechanism of action of each variable on the analysis objectives can be obtained, and the relevant results are summarized in Table 2.

From Table 2, it can be seen that increasing the yield strength and elastic modulus of the base material zone, heat-affected zone, and welded bead zone can all cause an increase in the maximum principal stress and maximum principal strain in the corresponding zones. In contrast, decreasing the yield strength and elastic modulus of the base material zone, heat-affected zone, and welded bead zone will lead to a reduction in the maximum principal stress and maximum principal strain in the corresponding zones, with the variation range ranging from 2% to 8%. Increasing the wheelset velocity can cause an increase in the maximum principal stress and maximum principal strain in the rail welded joint area; conversely, decreasing it will lead to a reduction in the maximum principal stress and maximum principal strain, with the variation range ranging from 1% to 5%. Unsprung mass has a significant influence on the maximum principal stress and maximum principal strain in the rail welded joint area, and there is a positive correlation between the two, with the variation range ranging from 7% to 11%. The wheel–rail interface friction coefficient also exhibits a positive correlation with the maximum principal stress and maximum principal strain in the rail welded joint area, with the variation range ranging from 2% to 3%. The primary suspension connection stiffness and damping, as well as the fastener stiffness and damping, have a relatively small influence on the maximum principal stress and maximum principal strain in the rail welded joint area, and all exhibit a positive correlation, with the variation range ranging from 0.01% to 0.6%.

As can be seen from the analysis in Table 2, improving the material properties of the heat-affected zone, such as increasing the yield strength and elastic modulus by 10%, has the most significant effect on reducing the maximum principal stress and strain in this region (i.e., improving the damage initiation conditions), with a variation range of 4–8%. This essentially verifies in reverse that material softening is the intrinsic cause for the susceptibility to damage in this region. Therefore, optimizing the welding process or heat treatment to improve the microstructure and mechanical properties of the heat-affected zone is the key to fundamentally enhancing the damage resistance of the joint. In terms of operating parameters, reducing the axle load (unsprung mass) can globally reduce the dynamic load input in the joint zone, thereby systematically decreasing the overall stress level of the joint with an influence amplitude of 11–12%. This makes it the primary external measure for controlling the dynamic response of rail welded joints. In contrast, the mechanism of reducing the friction coefficient is different. By reducing the tangential force transmission and slip dissipation, it directly alleviates the shear stress concentration and slip wear tendency in the surface layer, which is consistent with the slip contact characteristics revealed in Section 3.1. It is an effective regulation measure for interface damage. By comparison, varying the primary suspension connection parameters and fastener connection parameters leads to no obvious change in the maximum principal stress and maximum principal strain of the material in the welded joint zone. This indicates that, under the short time scale of heavy-load and high-frequency impact, the local stress–strain state of the joint zone is mainly dominated by the directly acting wheel–rail system (mass, friction) and the material’s own properties, and the parameters of the underlying infrastructure are not sensitive factors in such contact fatigue initiation problems.

It can be seen that to suppress plastic deformation and damage initiation of welded joints, corresponding measures should be taken in the priority order: improving material properties, regulating external loads, and optimizing interface conditions. This strategy provides a clear mechanical basis and engineering guidance for the performance optimization and maintenance decision-making of welded joints. It should be noted that although the parametric analysis demonstrates the systematic influence of friction coefficient variation on the contact mechanical state, the fluctuation of friction coefficient in practice is a time-varying result coupled with multiple factors such as environmental conditions, operating speed, and material surface status. In the future, it is necessary to establish a dynamic model that can reflect the constitutive relationship between environmental parameters (e.g., humidity, medium) and interface friction characteristics.

## 6. Conclusions

This study investigates the transient contact mechanical behavior of rail welded joints in heavy-haul railways via a validated 3D finite element model. First, a field investigation of typical railway lines was conducted, and based on the actual operation conditions of the line section, a 3D coupled numerical model of the vehicle wheelset and track was established. Second, the analysis of wheel–rail contact characteristics was carried out, including the stick-slip distribution characteristics and dynamic response characteristics. Third, the analysis of the elastic–plastic characteristics of the joint area under wheel–rail contact action was conducted, including the stress–strain time-varying features and spatial features. Finally, a systematic parameterization analysis was conducted to characterize the mechanism of action of influencing parameters on the system mechanical properties. The main conclusions obtained are as follows:

(1) When the wheel passes through the rail welded joint area, the wheel–rail contact changes from the stick-slip state corresponding to the base material zone to the slip state corresponding to the heat-affected zone and welded bead zone, and then changes back to the stick-slip state. This indicates that stick-slip motion occurs in the wheel during passage through the rail welded joint region. The occurrence of slip reflects that the surface material tends to undergo wear, and the wear range in the heat-affected zone and welded bead zone is relatively larger than that in the base material zone.
(2) The acceleration amplitude of the contact nodes in the base material zone is the largest, followed by that in the welded bead zone, and that in the heat-affected zone is the smallest, which is closely related to the material properties of the three zones. The stick-slip distribution characteristics and dynamic response characteristics indicate that instability has occurred in the wheel–rail system, and this instability will further induce the formation of damage on the wheel–rail surfaces.
(3) There is a possibility of plastic deformation occurring in the material on the contact surfaces of the base material zone, heat-affected zone, and welded bead zone of the rail welded joint. Both the maximum principal stress and maximum principal strain of the section where the contact node is located occur on the rail surface; therefore, the probability that rail damage first appears on the contact surface is higher.
(4) Increasing the yield strength and elastic modulus of the material in the rail welded joint area is conducive to controlling the occurrence of plastic deformation in the material; reducing the wheelset velocity, unsprung mass, and wheel–rail interface friction coefficient can effectively inhibit the tendency of plastic deformation. Changing the primary suspension connection parameters and fastener connection parameters has a relatively small effect on controlling the plastic deformation in the rail welded joint area.

In this study, the multi-mechanism coupling law of early damage initiation in rail welded joints of heavy-haul railways is revealed through refined numerical simulation, and the spatiotemporal coupling mechanism of material inhomogeneity, interface stick-slip and plastic strain accumulation is clarified. Through parametric analysis, the influence degrees of various engineering variables on damage initiation are quantified, providing a theoretical basis for joint performance design and life prediction. However, the model is based on typical parameters and simplified assumptions, and its quantitative prediction accuracy needs to be improved through comparison and verification with measured data. Future work will focus on introducing welding residual stress and real geometric morphology, calibrating the constitutive model combined with measured material properties, developing an environment-sensitive contact interface model, and establishing a defect-included propagation analysis model. In this way, the full-process prediction from damage initiation to propagation will be realized, and a service performance evaluation system closer to engineering practice will be constructed.

## Figures and Tables

**Table 1:** Model parameters.

Component		Parameters	Value
Unsprung mass and primary suspension		Unsprung mass [t]	11.5
		Connection stiffness [MN/m]	2
		Connection damping [kNs/m]	50
Rail/wheelset		Elastic modulus [GPa]	205
		Density [kg/m^3^]	7850
		Poisson’s ratio	0.3
		Yield strength [MPa]	752
Fastener		Vertical stiffness [MN/m]	80
		Longitudinal stiffness [MN/m]	40
		Lateral Stiffness [MN/m]	40
		Vertical Damping [kNs/m]	100
		Longitudinal Damping [kNs/m]	20
		Lateral Damping [kNs/m]	20
		Spacing/mm	600
Welded joint	Weld bead	Elastic modulus [GPa]	197
		Density [kg/m^3^]	7850
		Poisson’s ratio	0.28
		Yield strength [MPa]	702
	Heat-affected zone	Elastic modulus [GPa]	180
		Density [kg/m^3^]	7850
		Poisson’s ratio	0.25
		Yield strength [MPa]	655
Sleeper/subgrade		Yield strength [MPa]	36
		Density [kg/m^3^]	2600
		Poisson’s ratio	0.17

**Table 2:** Parametric calculation results (↑ indicates an increase, ↓ indicates a decrease).

Number	Analysis Variable	Variation Amplitude	Analysis Objective	
			Maximum Principal Stress [MPa]	Maximum Principal Strain
1	Yield strength of the base material zone [MPa]	10% ↑	4% ↑ (BMZ)	2% ↑ (BMZ)
		10% ↓	3% ↓ (BMZ)	2% ↓ (BMZ)
2	Yield strength of the heat-affected zone [MPa]	10% ↑	5% ↑ (HAZ)	3% ↑ (HAZ)
		10% ↓	4% ↓ (HAZ)	2% ↓ (HAZ)
3	Yield strength of the welded bead zone [MPa]	10% ↑	4% ↑ (WBZ)	3% ↑ (WBZ)
		10% ↓	3% ↓ (WBZ)	2% ↓ (WBZ)
4	Elastic modulus of the base material zone [GPa]	10% ↑	5% ↑ (BMZ)	4% ↑ (BMZ)
		10% ↓	5% ↓ (BMZ)	3% ↓ (BMZ)
5	Elastic modulus of the heat-affected zone [GPa]	10% ↑	8% ↑ (HAZ)	7% ↑ (HAZ)
		10% ↓	6% ↓ (HAZ)	6% ↓ (HAZ)
6	Elastic modulus of the welded bead zone [GPa]	10% ↑	6% ↑ (WBZ)	6% ↑ (WBZ)
		10% ↓	6% ↓ (WBZ)	5% ↓ (WBZ)
7	Wheelset velocity [km/h]	10% ↑	5% ↑	2% ↑
		10% ↓	4% ↓	1% ↓
8	Unsprung mass [t]	10% ↑	11% ↑	9% ↑
		10% ↓	12% ↓	7% ↓
9	Wheel–rail interface friction coefficient	10% ↑	3% ↑	2% ↑
		10% ↓	2% ↓	2% ↓
10	Primary suspension connection stiffness [MN/m]	10% ↑	0.5% ↑	0.3% ↑
		10% ↓	0.3% ↓	0.2% ↓
11	Primary suspension connection damping [kNs/m]	10% ↑	0.1% ↑	0.1% ↑
		10% ↓	0.1% ↓	0.1% ↓
12	Vertical stiffness of fastener [MN/m]	10% ↑	0.6% ↑	0.5% ↑
		10% ↓	0.6% ↓	0.6% ↓
13	Longitudinal stiffness of fastener [MN/m]	10% ↑	0.2% ↑	0.1% ↑
		10% ↓	0.1% ↓	0.1% ↓
14	Lateral stiffness of fastener [MN/m]	10% ↑	0.1% ↑	0.1% ↑
		10% ↓	0.1% ↓	0.1% ↓
15	Vertical damping of fastener [kNs/m]	10% ↑	0.3% ↑	0.2% ↑
		10% ↓	0.1% ↓	0.1% ↓
16	Longitudinal damping of fastener [kNs/m]	10% ↑	0.04% ↑	0.01% ↑
		10% ↓	0.03% ↓	0.01% ↓
17	Lateral damping of fastener [kNs/m]	10% ↑	0.03% ↑	0.01% ↑
		10% ↓	0.03% ↓	0.01% ↓

## Data Availability

The original contributions presented in this study are included in the article. Further inquiries can be directed to the corresponding author.

## References

[1] Yang L.S., Ouyang Q., Xue H.X. (2002). Study and practice of high-speed heavy rail weldability of Qinshen dedicated passenger line. J. Railw. Eng. Soc..

[2] Wang P.J. (2021). Research on Transient Impact Behavior of Wheel and Rail at Rail Welded Joints of High SPEED Railway. Master’s Thesis.

[3] Pang X., Kan Q.H., Zhao J.Z., Xu X., Zhu L.Q. (2019). Finite element analysis on three-dimensional wheel-rail rolling contact of U75V welded joint. J. Chengdu Univ. (Nat. Sci. Ed.).

[4] Xu K.Y., Wang Y., Lu X., Ding H.H., Cui X.L., Liu Q.Y., Wang W.J. (2025). Rolling wear and damage properties of three kinds of pearlite rail welded joints. Tribology.

[5] Yang D.X. (2012). Research on development direction of track technology of heavy-haul railway. J. Railw. Eng. Soc..

[6] Zhao X., Wen Z.F., Wang H.Y., Jin X.S. (2013). 3D transient finite element model for high-speed wheel-rail rolling contact and its application. J. Mech. Eng..

[7] Wang Z.Q., Lei Z.Y. (2023). Analysis of causes for rail corrugation on steel spring floating slab tracks of metro small radius curves. China Mech. Eng..

[8] Wang S., Wang K.Y., Huang C. (2013). Characteristics of wheel-rail dynamic interaction of heavy haul railways due to the rail welding joint. J. Chongqing Univ. Technol. (Nat. Sci.).

[9] (2019). Code for Design of Railway Track (Limit State Method).

[10] Fu P.P., Zhu Z.M. (2018). Finite element modeling and stress analysis of static bending tests for rail welded joints. Weld. Join..

[11] Li Y.B., Xie Z.Q., Mo C.L. (2005). Finite element analysis technology application to welding technology expert system: Welding technology expert system based on neural network I. Trans. China Weld. Inst..

[12] Romano S., Manenti D., Beretta S., Zerbst U. (2016). Semi-probabilistic method for residual lifetime of aluminothermic welded rails with foot cracks. Theor. Appl. Fract. Mech..

[13] Sarikavak Y., Turkbas O.S., Cogun C. (2020). Influence of welding on microstructure and strength of rail steel. Constr. Build. Mater..

[14] Ozakgul K., Piroglu F., Caglayan O. (2015). An experimental investigation on flash butt welded rails. Eng. Fail. Anal..

[15] Zhang H., Xie J.Q., Li C.A., Zhu Z.M. (2024). Modelling and numerical analysis for rotatory friction welding of U75V steel rails. Proc. Inst. Mech. Eng. Part L J. Mater.-Des. Appl..

[16] Cascino A., Meli E., Rindi A. (2025). Design and optimization of a hybrid railcar structure with multilayer composite panels. Materials.

[17] Tang J., Zhou Z., Chen H., Wang S., Gutiérrez A. (2022). Research on the lightweight design of GFRP fabric pultrusion panels for railway vehicle. Compos. Struct..

[18] Xiao H., Chen X., Zhao Y. (2022). Analysis of unilateral rail corrugation mechanism based on friction self-excited theory. J. Southwest Jiaotong Univ..

[19] Xiang P.C., Jiang W.J., Ding H.H., Wang W.J., Guo J., Liu Q.Y. (2021). Investigation on impact wear and damage properties of rail welded joints after two types of heat-treatments. Tribology.

[20] Xu Y.D., Zhou Y. (2003). Computer simulation analyses of rail welding joint. J. Tongji Univ. (Nat. Sci.).

[21] Song X.X., Zhang S.Y., Xu M.N., Ding H.H., Guo J., Zhao X., Qi H.F., Liu Q.Y., Wang W.J., Lewis R. (2025). Experimental investigation on dynamic adhesion characteristics of wheel-rail under various media conditions at a large slip ratio range. Wear.

[22] Su H., Pun C.L., Mutton P., Kan Q.H., Kang G.Z., Yan W.Y. (2021). Numerical study on the ratcheting performance of rail flash butt welds in heavy haul operations. Int. J. Mech. Sci..

[23] Su H., Pun C.L., Mutton P., Kan Q.H., Yan W.Y. (2019). Numerical study on the ratcheting performance of heavy haul rails in curved tracks. Wear.

[24] Wang Z.Q., Lei Z.Y. (2023). Mechanism of corrugation on the track with Cologne egg fasteners based on transient contact characteristics. J. Tsinghua Univ. (Sci. Technol.).

[25] Kou J., Zhang J.M., Zhou H.C., Wang C.P. (2021). Wheel-rail wear characteristics of intercity EMUs on curve in worn stages. J. Traffic Transp. Eng..

[26] Wang R.H., Jain V.K., Mall S. (2007). A non-uniform friction distribution model for partial slip fretting contact. Wear.

[27] Won H.I., Chung J. (2018). Numerical analysis for the stick-slip vibration of a transversely moving beam in contact with a frictional wall. J. Sound Vib..

[28] Chen J.J., Sun H.W., Jiao T., Liu Z.G., Xu B. (2020). Stick-slip vibration analysis and vibration frequency extraction of coke pushing system. Eng. Fail. Anal..

[29] Qu Y.G., Xie F.T., Su H., Meng G. (2021). Numerical analysis of stick-slip induced nonlinear vibration and acoustic responses of composite laminated plates with friction boundaries. Compos. Struct..

[30] Wang Z.Y., Wu B., Wu S., Wen Z.F. (2025). Effects of wheel tread hollow wear on wheel-rail adhesion under wet condition. Tribol. Int..

[31] Silva J.V.R.S.E., Strey N.F., dos Santos G.F.M., Scandian C. (2025). Wheel-rail wear severity prediction using semi-analytical computational method. Wear.

[32] Wen B.G., Tao G.Q., Wen Z.F. (2025). Prediction of locomotive wheel wear evolution considering thermo-mechanical coupling: Wear model and validation. Wear.

[33] Afferrante L., Ciavarella M. (2009). Corrugation models and the roaring rails enigma: A simple analytical contact mechanics model based on a perturbation of Carter’s solution. J. Mech. Mater. Struct..

[34] Barber J.R., Ciavarella M., Afferrante L., Sackfield A. (2008). Effect of small harmonic oscillations during the steady rolling of a cylinder on a plane. Int. J. Mech. Sci..

[35] Afferrante L., Ciavarella M. (2009). Short-pitch rail corrugation: A possible resonance-free regime as a step forward to explain the “enigma”?. Wear.

[36] Ciavarella M., Barber J. (2008). Influence of longitudinal creepage and wheel inertia on short-pitch corrugation: A resonance-free mechanism to explain the roaring rail phenomenon. Proc. Inst. Mech. Eng. Part J J. Eng. Tribol..

